# Aryl Hydrocarbon Receptor Signaling in Prostate Cancer Therapy: A Review of Implications for Anti-androgen Treatment Strategies and Resistance

**DOI:** 10.7759/cureus.65247

**Published:** 2024-07-24

**Authors:** Gurjot Singh, Shubam Trehan, Adarshpreet Singh, Kanishka Goswami, Amna Farooq, Priya Antil, Piyush Puri, Gaurav Bector, Aayush Jain, Waqas Azhar

**Affiliations:** 1 Internal Medicine, Southern Illinois University School of Medicine, Springfield, USA; 2 Internal Medicine, Memorial Medical Center, Springfield, USA; 3 Internal Medicine, Saint John Hospital, Springfield, USA; 4 Hospital Medicine, Springfield Clinic, Springfield, USA

**Keywords:** androgen antagonist, receptor signaling, anti androgen therapy, aryl hydrocarbon receptor, prostrate cancer

## Abstract

Prostate cancer is a leading cause of cancer-related morbidity and mortality in men, frequently exhibiting resistance to conventional anti-androgen therapies. This review investigates the emerging significance of the aryl hydrocarbon receptor (AhR) in prostate cancer, focusing on its role in modulating androgen receptor (AR) signaling and its potential as a therapeutic target. AhR, traditionally known for detoxifying harmful compounds, has been increasingly recognized for its dual capacity to either enhance or inhibit AR activity based on cellular context and specific coactivators. Furthermore, AhR influences tumor progression independently of AR by regulating genes involved in cell cycle control and apoptosis. This narrative review synthesizes current research on AhR’s multifaceted roles in prostate cancer, evaluates its potential as a biomarker, and discusses the therapeutic implications of targeting AhR, particularly for hormone-refractory prostate cancer. Our findings underscore the necessity for personalized AhR-targeted therapies and advocate for continued clinical research to fully leverage AhR's therapeutic potential.

## Introduction and background

Prostate cancer remains a significant global health concern, being the second most commonly diagnosed cancer among men worldwide and the most frequently diagnosed in men in the United States, excluding skin cancers [1]. The incidence of prostate cancer varies significantly across different regions, influenced by genetic, environmental, and lifestyle factors [2]. For instance, the incidence rates in Northern Europe and North America are notably higher compared to Asian and African countries, reflecting differences in screening practices, dietary habits, and genetic predispositions [3].

Epidemiological studies estimate that approximately one in eight men will be diagnosed with prostate cancer during their lifetime, highlighting the substantial public health impact of this disease [4]. The disease predominantly affects older men, with the average age of diagnosis being around 66 years. Despite advances in screening and early detection, prostate cancer remains a leading cause of cancer-related morbidity and mortality among men, with an estimated 34,700 men expected to die from the disease in 2023 [1]. The relative five-year survival rate for localized prostate cancer is nearly 100%, but this rate drops to around 30% for men with distant metastasis, underscoring the severity of advanced-stage disease [4].

Androgen deprivation therapy (ADT) has long been the cornerstone of treatment for advanced prostate cancer. This therapeutic approach reduces levels of circulating androgens, primarily testosterone, which are essential for the growth and survival of prostate cancer cells [5]. Despite the initial efficacy of ADT, most patients eventually develop resistance, leading to a more aggressive form of the disease known as castration-resistant prostate cancer (CRPC) [6]. CRPC is characterized by the ability of prostate cancer cells to proliferate despite castrate levels of testosterone. This resistance to ADT is a major clinical challenge, as it signifies the progression of the disease to a more lethal state.

Various mechanisms contribute to the development of androgen resistance, including androgen receptor (AR) overexpression, AR gene mutations, and the activation of alternative signaling pathways that bypass the need for androgens [7]. Studies have shown that alterations in AR coregulators and coactivators play a crucial role in maintaining AR signaling in the absence of androgens. For instance, AR splice variants that lack the ligand-binding domain (LBD) but remain constitutively active have been identified in CRPC, contributing to sustained AR signaling and tumor growth. Furthermore, intratumoral androgen synthesis has been observed in CRPC, allowing cancer cells to produce their own androgens and sustain AR activation [8].

CRPC continues to grow and spread despite achieving castrate levels of testosterone through ADT. CRPC is a particularly aggressive and deadly form of prostate cancer, with limited treatment options and a poor prognosis. The median survival for patients with CRPC is approximately two to three years from the time of diagnosis, highlighting the urgent need for new therapeutic strategies [9]. CRPC can manifest as either a non-metastatic or metastatic disease. Non-metastatic CRPC (mCRPC) is characterized by rising prostate-specific antigen (PSA) levels without radiographic evidence of metastasis, while mCRPC involves the spread of cancer to distant sites such as bones and visceral organs [10]. The management of CRPC requires a multidisciplinary approach, incorporating novel hormonal agents, chemotherapy, immunotherapy, and targeted therapies.

Recent advancements in the understanding of CRPC have led to the development of several new therapeutic agents. For example, second-generation anti-androgens such as enzalutamide and apalutamide have been shown to significantly improve survival in patients with CRPC by more effectively inhibiting AR signaling [11-12]. Additionally, androgen biosynthesis inhibitors like abiraterone acetate target the enzyme CYP17A1, which is involved in androgen production, thereby reducing intratumoral androgens and inhibiting tumor growth [13]. The emergence of resistance to these new agents, however, remains a significant challenge. Mechanisms of resistance include further mutations in the AR gene, activation of glucocorticoid receptors (GRs), and alterations in DNA repair pathways [14]. Understanding these resistance mechanisms is critical for developing next-generation therapies that can overcome resistance and improve outcomes for patients with CRPC.

Emerging research has highlighted the aryl hydrocarbon receptor (AhR) as a potential target in the fight against prostate cancer. Traditionally known for its role in detoxifying environmental toxins and xenobiotics, AhR has been implicated in a variety of cellular processes, including cell proliferation, differentiation, and immune response modulation [15]. In the context of prostate cancer, AhR plays a complex role, capable of both promoting and inhibiting tumor growth depending on the cellular environment and the presence of specific coactivators and corepressors.

Activation of AhR with certain ligands has been shown to enhance AR-mediated transcription and promote tumor cell proliferation [16]. Conversely, AhR can also inhibit AR signaling by recruiting corepressors or promoting AR degradation, thus suppressing tumor growth [17]. This dual functionality makes AhR a compelling target for therapeutic intervention, particularly in CRPC where traditional therapies have failed. Moreover, AhR's involvement in regulating immune responses and inflammatory pathways adds another layer of therapeutic potential. By modulating the tumor microenvironment, AhR-targeted therapies could enhance anti-tumor immune responses and improve the efficacy of existing treatments [18]. Given the complexity of AhR's role in prostate cancer, further research is necessary to fully elucidate its mechanisms and develop effective AhR-targeted therapies.

This review aims to synthesize current research on the role of AhR in prostate cancer, exploring its potential as a therapeutic target and biomarker. By addressing the challenges and future directions in this field, we hope to shed light on the promising avenues for improving outcomes for patients with advanced prostate cancer.

## Review

Pathophysiology and molecular mechanisms

**Figure 1:**
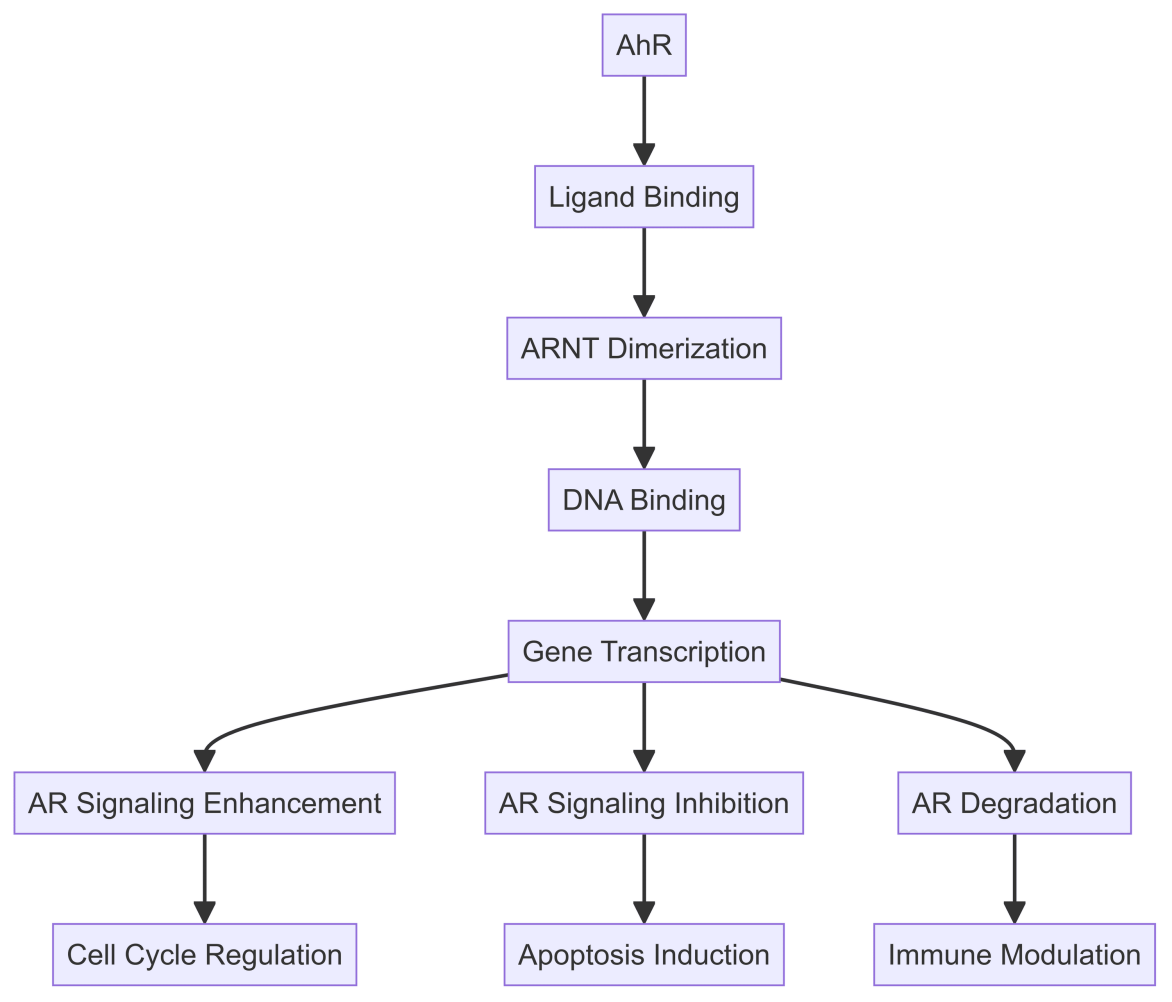
Flowchart presenting molecular mechanisms of the AhR receptor in prostate cancer Image Credits: Shubam Trehan

The AR signaling pathway is integral to the development and progression of prostate cancer. AR, a nuclear hormone receptor, regulates the expression of genes involved in prostate cell growth and survival. Androgens, mainly testosterone and dihydrotestosterone (DHT), initiate the pathway by binding to AR, facilitating its translocation to the nucleus, where it alters gene expression [19]. In prostate cancer, the AR signaling pathway is often upregulated, leading to uncontrolled cell proliferation. Key steps in this pathway include ligand binding and AR activation, where androgens bind to the LBD of AR, causing a conformational change that activates AR. This activated AR translocates into the nucleus [20]. Once in the nucleus, AR binds to androgen response elements (AREs) in the promoter regions of target genes. This binding recruits coactivators and other transcriptional machinery to initiate gene transcription [21]. Genes regulated by AR include those involved in cell cycle progression, survival, and proliferation (Figure 1).

Crosstalk with other pathways

AR signaling also interacts with other pathways, such as PI3K/AKT, MAPK, and Wnt/β-catenin, which can modulate its activity and contribute to prostate cancer progression. Crosstalk between these pathways and AR signaling can enhance the growth and survival of prostate cancer cells [22]. Recent studies have highlighted the inhibitory crosstalk between AhR and AR in prostate cancer. For example, AhR activation can lead to the ubiquitination and degradation of AR, reducing its transcriptional activity and potentially inhibiting tumor growth [14,23-26]. This mechanism suggests a therapeutic potential for AhR agonists or antagonists in targeting AR signaling pathways. Furthermore, AhR has been shown to interact with other signaling pathways such as PI3K/AKT, which further influences prostate cancer progression [27]. The interaction between AhR and other signaling pathways, such as PI3K/AKT, adds another layer of complexity to prostate cancer progression. For instance, AhR activation has been linked to the upregulation of RAB3D, which promotes prostate cancer progression through the PI3K/AKT pathway [27]. Additionally, AhR interacts with other pathways, such as MAPK and Wnt, which further modulate AR activity. These interactions contribute to prostate cancer progression by enhancing cell proliferation, survival, and migration. Specifically, the activation of the MAPK pathway by AhR can lead to increased AR signaling, while cross-talk with the Wnt pathway can influence gene expression profiles associated with tumor growth and metastasis. Understanding these detailed mechanisms provides deeper insights into how these pathways collectively contribute to the advancement of prostate cancer.

Mechanisms of resistance

Despite the initial effectiveness of anti-androgen therapies, resistance often develops, leading to CRPC. Mechanisms of resistance include AR gene amplification and overexpression, where cancer cells become more sensitive to low androgen levels due to an increased number of AR gene copies or higher AR protein levels. AR mutations can alter the receptor’s ligand specificity, allowing it to be activated by other steroids or the anti-androgens themselves [23]. Splice variants of AR, such as AR-V7, lack the LBD but remain constitutively active, driving androgen-independent growth. Additionally, activation of bypass signaling pathways, such as the glucocorticoid receptor (GR) and neuroendocrine differentiation (NED), can sustain tumor growth independent of AR signaling [24]. The activation of bypass signaling pathways plays a critical role in the development of treatment resistance in prostate cancer. When AR signaling is inhibited by therapies, cancer cells can adapt by activating alternative pathways that allow them to survive and proliferate. One such pathway involves the GR, which can take over the role of AR in promoting cancer cell growth. Studies have shown that GR expression increases in some prostate cancer cells following treatment with AR inhibitors, enabling these cells to bypass AR blockade and continue to grow.

Another significant mechanism is NED, where prostate cancer cells lose their dependence on AR signaling and gain characteristics of neuroendocrine cells. This transition is often driven by the activation of lineage plasticity and reprogramming pathways, allowing the cancer to adopt a more aggressive phenotype that is resistant to standard AR-targeted therapies. NED is marked by the expression of neuroendocrine markers and is associated with a worse prognosis.

Understanding these mechanisms of resistance is crucial for developing new therapeutic strategies to overcome or prevent resistance in prostate cancer. Targeting the bypass pathways and the specific alterations that drive resistance, such as GR and neuroendocrine differentiation, offers potential avenues for improving treatment outcomes in patients with CRPC.

Role of AhR in AR signaling

The AhR adds another layer of complexity to this signaling network. Traditionally known for its role in xenobiotic metabolism, AhR has been implicated in various cellular processes, including cell proliferation, differentiation, and immune response modulation [25]. In prostate cancer, AhR interacts with AR signaling in a context-dependent manner, exhibiting both enhancement and inhibition of AR activity. In some contexts, AhR can act as a coactivator of AR, enhancing AR-mediated transcription by recruiting coactivators to the AR complex and promoting the activation of AR target genes involved in cell growth and survival [14,26]. This dual role of AhR is highlighted in studies where its overexpression enhances Src kinase activity, thereby promoting AR signaling and prostate cancer progression [16]. Conversely, AhR can inhibit AR signaling through the recruitment of corepressors or the ubiquitin-proteasome pathway, accelerating AR degradation and reducing its transcriptional activity, potentially inhibiting tumor growth. The presence of specific coactivators, such as FHL2, can further determine whether AhR enhances or inhibits AR activity [26]. Additionally, AhR controls genes that regulate the cell cycle and apoptosis, influencing tumor growth independently of AR [27].

AhR interactions with the immune system

AhR also plays a significant role in modulating the immune system within the tumor microenvironment, influencing prostate cancer progression [16]. AhR activation can alter the balance of immune cell populations and their function. For example, AhR activation in dendritic cells can promote the differentiation of regulatory T cells (Tregs), which are known to suppress anti-tumor immune responses and promote tumor growth. Conversely, AhR activation in T cells can lead to the production of anti-inflammatory cytokines, such as IL-10, further promoting an immunosuppressive environment. In some contexts, AhR can also influence the differentiation and function of myeloid-derived suppressor cells (MDSCs), which are potent immunosuppressive cells that inhibit the activity of cytotoxic T lymphocytes and natural killer (NK) cells. Thus, the interaction of AhR with various components of the immune system can either promote or inhibit prostate cancer progression, depending on the specific cellular and molecular context.

Discussion

The Dual Role of AhR in Prostate Cancer Progression

The AhR plays a multifaceted role in the progression of prostate cancer, demonstrating both tumor-promoting and tumor-suppressing activities. This dual functionality is highly dependent on the cellular context and the presence of various coactivators and corepressors (Table 1). Studies have shown that AhR can enhance AR activity, thereby promoting tumor growth [8]. For instance, in vitro studies using prostate cancer cell lines have demonstrated that activation of AhR with ligands such as dioxins and certain polycyclic aromatic hydrocarbons leads to increased AR transcriptional activity and tumor cell proliferation. A study illustrated that AhR overexpression enhances Src kinase activity, which in turn promotes AR signaling and prostate cancer progression. This study found that AhR acts as a coactivator of AR in the presence of specific ligands, facilitating AR-mediated transcription of genes involved in cell proliferation and survival [27]. Another significant study provided evidence that AhR activation with specific ligands increases AR transcriptional activity, leading to enhanced tumor cell proliferation. This research emphasized AhR's role as a coactivator of AR, highlighting its potential in promoting prostate cancer progression. The overexpression of AhR was shown to enhance Src kinase activity, further promoting AR signaling and prostate cancer progression [16]. These findings suggest that AhR can act as a significant promoter of prostate cancer under specific conditions, contributing to tumor growth and progression.

**Table 1:** The table highlights the key coactivators and corepressors involved in AhR’s dual functionality in prostate cancer, demonstrating their roles in either promoting or suppressing tumor activity.

Function	Coactivators/corepressors	Role in prostate cancer
Tumor-promoting activity	ARNT (aryl hydrocarbon receptor nuclear translocator)	Forms a complex with AhR, activates transcription of genes involved in cell proliferation and survival
	SRC-1 (steroid receptor coactivator-1)	Enhances AhR-mediated transcriptional activity, promoting tumor growth
	p300/CBP (CREB binding protein)	Facilitates chromatin remodeling, enhances transcription of AhR target genes
	Cyclin D1	Upregulated by AhR, promotes cell cycle progression and proliferation
	β-Catenin	Activates Wnt signaling pathway promotes cell proliferation and metastasis
	NF-κB (nuclear factor kappa-light-chain-enhancer of activated B cells)	Modulates NF-κB signaling, contributing to inflammation and cancer progression
Tumor-suppressing activity	TLE1 (transducin-like enhancer of split 1)	Acts as a corepressor of AhR, suppressing genes involved in cell proliferation
	HDACs (histone deacetylases)	Remove acetyl groups from histones, leading to chromatin condensation and suppression of AhR target genes
	SMRT (silencing mediator for retinoid and thyroid receptors)	Represses AhR-mediated transcriptional activity, reducing expression of tumor-promoting genes
	NCoR (nuclear receptor corepressor)	Inhibits AhR activity, promoting tumor-suppressive gene expression
	RB (retinoblastoma protein)	Interacts with AhR to inhibit cell cycle progression, contributing to tumor suppression
	BRCA1 (breast cancer 1)	Interacts with AhR, suppresses DNA damage response genes, promotes apoptosis

Conversely, AhR can inhibit AR signaling under different conditions. This inhibitory effect is often mediated through the recruitment of corepressors or by promoting the degradation of AR. Research indicates that in the presence of the coactivator FHL2, AhR can recruit repressive complexes to the AR signaling axis, thereby diminishing AR-mediated transcription [28]. Activation of AhR has also been linked to the ubiquitination and proteasomal degradation of AR, which further reduces its function. For example, one study found that the AhR antagonist carbidopa promotes the ubiquitination and degradation of AR, leading to a significant reduction in AR protein levels [16]. This degradation mechanism, mediated by AhR, resulted in the suppression of prostate cancer cell proliferation, with pronounced effects on hormone-refractory prostate cancer cell lines. Additionally, Ghotbaddini et al. [22] explored how AhR activation leads to AR degradation via the ubiquitin-proteasome pathway, further reducing AR function in prostate cancer cells. This study highlighted the dual role of AhR in prostate cancer progression, suggesting that the specific molecular and cellular environment impacts AhR's effect on tumor progression. The complexity of AhR's role in prostate cancer underscores the necessity for context-dependent therapeutic strategies targeting AhR.

AhR as a Biomarker for Prostate Cancer Aggressiveness

AhR's activity levels have been associated with the aggressiveness of prostate cancer, suggesting its potential as a prognostic biomarker. Higher AhR activity has been observed in more advanced and aggressive forms of prostate cancer, indicating that AhR could be used to stratify patients based on the likely progression of their disease. A study utilized immunohistochemical analysis to assess AhR levels in prostate cancer tissue samples [23]. The results demonstrated a significant correlation between high AhR expression and higher Gleason scores, which are indicative of more aggressive and poorly differentiated tumors. By integrating AhR activity measurements into clinical practice, oncologists could better predict which patients are likely to experience rapid disease progression and might benefit from more aggressive treatment regimens.

Additionally, monitoring AhR levels during treatment could help assess the effectiveness of therapeutic interventions and may detect early signs of treatment resistance. Another study by Novikov et al. [24] demonstrated the association of AhR activity levels with the aggressiveness of prostate cancer, supporting its use as a prognostic biomarker. The use of AhR as a biomarker for prostate cancer aggressiveness could provide valuable prognostic information, helping to stratify patients and tailor treatment approaches based on individual tumor characteristics.

Therapeutic Implications of AhR in Hormone-Refractory Prostate Cancer

Recent studies have explored the potential of AhR antagonists and agonists in treating hormone-refractory prostate cancer. Chen et al. [19] and Zgarbová and Vrzal [23] demonstrated that AhR antagonists like carbidopa and natural AhR agonists, respectively, can effectively reduce AR activity and prostate cancer cell viability, highlighting the potential for these compounds in therapeutic applications. Expanding on this, Griffith and Frankel [29] provided a comprehensive review of current drugs in development that target AhR. The review summarized the preclinical efficacy of various AhR modulators, showing promising results in reducing prostate cancer cell viability and inducing apoptosis (Table 2). These AhR-targeted compounds include both natural and synthetic agonists, which activate AhR to induce apoptosis, and antagonists, which inhibit AR signaling and promote AR degradation [3].

**Table 2:** Tabulated form of various AhR modulators and their mechanisms involved in reducing prostate cancer cell viability and inducing apoptosis.

AhR modulator	Mechanism of action
TCDD (tetrachlorodibenzo para dioxin)	Binds to AhR, translocates to the nucleus, and alters gene expression, leading to apoptosis
DIM (diindolylmethane)	Inhibits cell proliferation and induces apoptosis via modulation of cell cycle regulators
ITE (2-(1H-Indol-3-yl-carbonyl)-thiazole-4-carboxylic acid methyl ester)	Binds to AhR, induces apoptosis, and inhibits cell proliferation
Resveratrol	Antagonizes AhR modulates gene expression and induces cell cycle arrest and apoptosis
Flavonoids	Modulates AhR activity, leading to reduced cell viability and increased apoptosis
BaP (benzo[a]pyrene)	Activates AhR, leads to the expression of cytochrome P450 enzymes, causing oxidative stress and apoptosis
Curcumin	Modulates AhR signaling, reduces cell viability, induces apoptosis through mitochondrial pathways
Quercetin	Inhibits AhR-mediated transcription, induces apoptosis and cell cycle arrest
FICZ (6-formylindolo[3,2-b]carbazole)	Activates AhR, induces apoptosis and reduces cell viability through modulation of target genes
Kynurenine	Binds to AhR, alters immune response, induces apoptosis

Natural AhR agonists, such as indoles found in cruciferous vegetables, have been shown to effectively reduce prostate cancer cell viability and induce apoptosis by altering gene expression involved in cell cycle regulation and apoptosis [10]. Synthetic AhR agonists have also demonstrated significant preclinical efficacy in reducing tumor growth by selectively activating AhR in prostate cancer cells, thereby minimizing off-target effects [3]. Carbidopa, an AhR antagonist, has been shown to promote the ubiquitination and proteasomal degradation of AR, leading to a significant reduction in AR protein levels and decreased prostate cancer cell viability [9]. Other AhR antagonists have similarly demonstrated their ability to block AR activity and reduce tumor growth, providing a robust strategy for targeting hormone-refractory prostate cancer [3]. Additionally, selective AhR modulators (SAhRMs) have shown promise in preclinical models by selectively modulating AhR activity to induce apoptosis and reduce cell viability. These modulators offer a targeted approach that can be customized based on the specific needs of the patient and the characteristics of the tumor [3].

In support of these findings, Devlies et al. [30] conducted a preclinical evaluation of novel AhR antagonists in prostate cancer models. The study demonstrated that these antagonists significantly reduced prostate cancer cell viability and tumor growth in both in vitro and in vivo models. The AhR antagonists induced apoptosis by downregulating AhR target genes involved in cell proliferation and survival and interfering with the interaction between AhR and AR signaling. These results underscore the potential of AhR antagonists as effective therapeutic agents for prostate cancer, particularly in cases of hormone-refractory or advanced disease [4]. Furthermore, De Bono et al. [6] provided evidence for the synergistic effects of combining AhR modulators with standard chemotherapy agents. Their study showed that this combination significantly enhanced the efficacy of chemotherapy in prostate cancer models, reducing cell viability and inducing apoptosis to a greater extent than either treatment alone. This synergistic effect was observed across different prostate cancer cell lines and in vivo models, suggesting broad applicability. The combination therapy also helped overcome chemotherapy resistance, particularly in androgen-independent prostate cancer models, restoring sensitivity to chemotherapy and improving treatment outcomes [5].

Additionally, Khamit-Kush et al. [31] explored the epigenetic regulation of AhR in prostate cancer and its therapeutic implications. Their study found that epigenetic modifications, such as DNA methylation and histone modifications, regulate AhR expression and activity. The research demonstrated that epigenetic drugs, including DNA methyltransferase inhibitors and histone deacetylase inhibitors, could restore AhR expression and activity, leading to reduced tumor cell viability and enhanced apoptosis. These findings suggest that targeting the epigenetic regulation of AhR could be a novel therapeutic strategy for treating aggressive prostate cancer. Combining AhR-targeted therapies with epigenetic drugs could further enhance treatment efficacy [28].

Challenges and future directions

While the potential of AhR as a therapeutic target in prostate cancer is promising, several challenges remain. The dual role of AhR in modulating AR signaling complicates therapeutic strategies, necessitating personalized approaches based on the unique characteristics of each patient's tumor. Additionally, the context-dependent effects of AhR require further investigation to fully understand the mechanisms underlying its bidirectional influence on tumor progression. Future research should focus on elucidating the molecular mechanisms by which AhR interacts with AR and other signaling pathways in different cellular contexts. Some studies emphasized the necessity for more detailed mechanistic studies to fully understand AhR's role in prostate cancer. Additionally, the development and testing of novel AhR modulators, both antagonists and agonists, in preclinical and clinical settings are essential for advancing AhR-targeted therapies [32-33].

Exploring combination therapies that target AhR alongside other pathways critical for tumor growth and survival is another important direction for future research. Many studies have discussed the interactions between AR signaling and other molecular pathways in prostate cancer progression, highlighting the potential for combination therapies to enhance treatment efficacy and overcome resistance mechanisms [27,29-33]. Investigating the potential of AhR as a biomarker for early detection of treatment resistance and disease progression is also crucial [24]. Some studies have demonstrated the association of AhR activity levels with prostate cancer aggressiveness, supporting its use as a prognostic biomarker [22,29]. Further research is needed to validate these findings and develop reliable assays for measuring AhR activity in clinical settings.

## Conclusions

AhR represents a multifaceted and promising target in prostate cancer therapy. Given AhR's ability to modulate AR signaling bidirectionally and its independent effects on gene transcription and tumor cell proliferation, it holds potential as both a therapeutic target and a biomarker for disease aggressiveness. While significant challenges remain, ongoing research and clinical trials offer the potential to unlock the full therapeutic value of AhR-targeted treatments, providing new hope for patients with hormone-refractory prostate cancer.
